# The Polyamine Putrescine Contributes to H_2_O_2_ and *RbohD/F*-Dependent Positive Feedback Loop in *Arabidopsis* PAMP-Triggered Immunity

**DOI:** 10.3389/fpls.2019.00894

**Published:** 2019-07-16

**Authors:** Changxin Liu, Kostadin E. Atanasov, Antonio F. Tiburcio, Rubén Alcázar

**Affiliations:** Department of Biology, Healthcare and Environment, Section of Plant Physiology, Faculty of Pharmacy, University of Barcelona, Barcelona, Spain

**Keywords:** polyamines, putrescine, defense, pathogen-associated molecular pattern, reactive oxygen species, PAMP-triggered immunity

## Abstract

Polyamines are involved in defense against pathogenic microorganisms in plants. However, the role of the polyamine putrescine (Put) during plant defense has remained elusive. In this work, we studied the implication of polyamines during pathogen-associated molecular pattern (PAMP)-triggered immunity (PTI) in the model species *Arabidopsis thaliana*. Our data indicate that polyamines, particularly Put, accumulate in response to non-pathogenic *Pseudomonas syringae* pv. *tomato* DC3000 *hrcC* and in response to the purified PAMP flagellin22. Exogenously supplied Put to *Arabidopsis* seedlings induces defense responses compatible with PTI activation, such as callose deposition and transcriptional up-regulation of several PTI marker genes. Consistent with this, we show that Put primes for resistance against pathogenic bacteria. Through chemical and genetic approaches, we find that PTI-related transcriptional responses induced by Put are hydrogen peroxide and NADPH oxidase (*RBOHD* and *RBOHF*) dependent, thus suggesting that apoplastic ROS mediates Put signaling. Overall, our data indicate that Put amplifies PTI responses through ROS production, leading to enhanced disease resistance against bacterial pathogens.

## Introduction

To face against biotic stress, plants have evolved complex and effective defense systems (Dodds and Rathjen, 2010). A first barrier of plant defense is the presence of the cuticle and the cell wall, which act as physical barriers (Yeats and Rose, 2013). However, when pathogens break these preformed barriers, sophisticated mechanisms of pathogen recognition are involved (Bigeard et al., 2015). Plasma membrane pathogen or pattern recognition receptors (PRRs) recognize pathogen-associated molecular patterns (PAMPs) that lead to PAMP-triggered immunity (PTI) (Zipfel and Felix, 2005; Iakovidis et al., 2016). One of the most well-characterized PAMPs is flagellin, a structural component of the flagellum in Gram-negative bacteria. The peptide flagellin22 (flg22) is recognized by the leucine-rich repeat receptor kinase FLS2 (FLAGELLIN SENSING 2) (Felix et al., 1999; Gómez-Gómez and Boller, 2002). Known responses to PTI are the generation of reactive oxygen species (ROS), cell wall reinforcement by callose deposition, and changes in the expression of defense-related genes (Boller and Felix, 2009; Nicaise et al., 2009; Ahuja et al., 2012). ROS production inhibits pathogen growth, stimulates cell wall cross-linking, and mediates the signal transduction for transcriptional changes (Apel and Hirt, 2004). NADPH oxidases are membrane-bound enzymes important for the generation of ROS during biotic and abiotic stresses, growth, and development. They transfer electrons from cytosolic NADPH or NADH to apoplastic oxygen, producing anion superoxide O_2_^−^ in the apoplast, which can be converted to hydrogen peroxide (H_2_O_2_) by superoxide dismutase (Kadota et al., 2015). *Arabidopsis thaliana* (*Arabidopsis*) carries 10 genes encoding NADPH oxidases, which belong to the *RBOH* (*RESPIRATORY BURST OXIDASE HOMOLOG*) family. Among them, RBOHD and, to a lesser extent, RBOHF are required for the generation of apoplastic ROS during incompatible plant–pathogen interactions (Torres et al., 2002). *RBOHD* is required for cell death control, cell wall damage-induced lignification, and systemic signaling in response to biotic and abiotic stresses (Torres et al., 2005; Miller et al., 2009; Denness et al., 2011). RBOHD and RBOHF fine-tune the spatial control of ROS production and hypersensitive response (HR) in and around infection sites (Torres et al., 2002, 2005, 2006; Chaouch et al., 2012). In addition to NADPH oxidases, apoplastic ROS can also be originated from polyamine oxidation. Polyamines are small polycationic molecules bearing amino groups. Most abundant plant polyamines are putrescine (Put), spermidine (Spd), and spermine (Spm), and they can be found in free forms or conjugated to hydroxycinnamic acids. Polyamines accumulate in response to different abiotic and biotic stresses and can be oxidatively deaminated by amine oxidases generating H_2_O_2_ (Tiburcio et al., 2014). Based on the cofactor involved, amine oxidases are classified in copper-containing amine oxidases (CuAOs) and FAD-dependent polyamine oxidases (PAOs). CuAOs catalyze the oxidation of Put at its primary amino group, producing 4-aminobutanal along with H_2_O_2_ and NH_4_^+^ (Cona et al., 2006; Angelini et al., 2010). In *Arabidopsis*, PAOs are involved in back-conversion reactions that convert Spm, thermospermine (tSpm), and Spd in their immediate precursors, producing 3-aminopropanal and H_2_O_2_ (Moschou et al., 2012; Ono et al., 2012; Ahou et al., 2014; Kim D.W et al., 2014). Some amine oxidases are located in the apoplast and may function as a source for apoplastic H_2_O_2_ during the elicitation of plant defense. For instance, inoculation of tobacco plants carrying the *N* resistance gene with tobacco mosaic virus (TMV) triggers HR and the accumulation of Spm in the apoplast (Yamakawa et al., 1998). In this species, Spm activates mitogen-activated protein kinases (MAPKs) SIPK (SA-induced protein kinase) and WIPK (wound-induced protein kinase) (Zhang and Klessig, 1997; Seo et al., 2007) and induces changes in the expression of Spm-responsive genes, some coding for acidic pathogenesis-related proteins (Yamakawa et al., 1998). Also in tobacco, inoculation with the hemibiotrophic bacteria *Pseudomonas viridiflava* and *Pseudomonas syringae* pv. *tabaci* leads to increases in Spm levels in the apoplast, which associate with disease resistance compromised by PAO inhibitors (Marina et al., 2008; Moschou et al., 2009). In *Arabidopsis*, Spm and its isomer tSpm trigger transcriptional changes that restrict the multiplication of cucumber mosaic virus (CMV) (Mitsuya et al., 2009; Sagor et al., 2012). Also in this species, transgenic plants that accumulate Spm by overexpression of *SAMDC1* (*S-ADENOSYLMETHIONINE DECARBOXYLASE 1*) or *SPMS* (*SPERMINE SYNTHASE*) exhibit enhanced disease resistance against *P. syringae* pv. *maculicola* ES4326, *P. syringae* pv. *tomato* DC3000 (*Pst* DC3000), and *P. viridiflava* (Gonzalez et al., 2011; Marco et al., 2014). Overall, the polyamine Spm seems important for the establishment of HR and basal defense responses to hemibiotrophic pathogens in tobacco and *Arabidopsis*. Conversely, Put has not been observed to have such defense-promoting activities, although its content is remarkably increased in response to pathogens (Yoda et al., 2003; Mitsuya et al., 2009; Sagor et al., 2012; Vilas et al., 2018; Seifi and Shelp, 2019).

In this work, we studied the involvement of polyamines during PTI in *Arabidopsis*. We report that Put accumulates in response to inoculation with the type three secretor system (TTSS) defective *P. syringae* DC3000 *hrcC* mutant strain (*hrcC*), which induces a strong PTI response (Yuan and He, 1996; Tsuda et al., 2008), and this accumulation is not suppressed by *Pst* DC3000 type III effectors (Cunnac et al., 2009). Consistent with a potential role for Put during PTI, we show that this polyamine also accumulates in response to flg22, one of the most well-characterized PAMPs. Through the analysis of *arginine decarboxylase 1* (*adc1*) and *arginine decarboxylase 2* (*adc2*) loss-of-function mutants, deficient in Put biosynthesis, we find that the *ADC2* isoform is the major contributor to Put biosynthesis triggered by flg22. We show that Put induces the formation of callose deposits, a typical response of PTI, when applied to *Arabidopsis* seedlings. In addition, we demonstrate that Put quickly induces the expression of several PTI marker genes (Huffaker and Ryan, 2007; Xiao et al., 2007; Denoux et al., 2008; Wang et al., 2009; Boudsocq et al., 2010; Cheng et al., 2013; Po-Wen et al., 2013; Shi et al., 2015), and these transcriptional changes are compromised in the presence of the H_2_O_2_ scavenger dimethylthiourea (DMTU), and in *atrbohD*, *atrbohF*, and double *atrbohD/F* NADPH oxidase loss-of-function mutants. We finally report that Put can be regarded as a priming agent that contributes to basal disease resistance against bacterial pathogens. Overall, we provide evidence that Put contributes to H_2_O_2_ and *RBOHD/F*-dependent positive feedback loop amplification of PTI.

## Materials and Methods

### Plant Materials and Growth Conditions

Plants were grown on soil (peat moss:vermiculite:perlite, 40:50:10) at 20–22°C under 12-h dark/12-h light cycles at 100–125 μmol photons m^–1^ s^–2^ of light intensity and 70% relative humidity. For *in vitro* culture, seeds were sterilized with a solution containing 30% sodium hypochlorite supplemented with 0.5% Triton X-100 for 10 min, followed by three washes with sterile distilled H_2_O. Sterilized seeds were sown on growth media [GM, 1/2 Murashige and Skoog supplemented with vitamins, 1% sucrose, 0.6% plant agar (Duchefa Biochemie), and pH 5.7 adjusted with 1 M KOH]. Plates were kept in the dark at 4°C for stratification for 2–3 days. Seedlings were grown under 12-h dark/12-h light cycles at 20–22°C, 100–125 μmol photons m^–2^ s^–1^ of light intensity. flg22 peptide was purchased from Anaspec^1^. The *fls2* mutant was kindly provided by Jane Parker (Zipfel et al., 2004). The *adc1-2* (SALK_085350), *adc2-4* (SALK_147171), *atrbohD* (SALK_109396 and SALK_005253), *atrbohF* (SALK_044584 and SALK_077748), and double *atrbohD/F* (N9558) (Torres et al., 2002) mutants were obtained from the Nottingham *Arabidopsis* Stock Center^2^. The *adc1-3* and *adc2-3* mutants were previously reported (Cuevas et al., 2008). The *gsl5* mutant was kindly provided by Christian Voigt.

### Polyamine Levels Determination

Polyamines were derivatized with dansyl chloride and analyzed by high-performance liquid chromatography (HPLC) as previously described (Marcé et al., 1995; Zarza et al., 2017). All harvested tissues were washed three times with sterile distilled H_2_O before processing or freezing in liquid nitrogen. Apoplastic polyamines were determined according to Yoda et al. (2009). All polyamine analyses were performed in at least three biological replicates.

### Histochemical Analyses

For aniline blue staining, seedlings were fixed and cleared in a solution of acetic acid/ethanol (1:3) overnight, followed by two washes of 30 min in 150 mM K_2_HPO_4_ and staining with 0.01% aniline blue (Sigma) for 2 h in the same buffer. Observations were performed under an epifluorescence microscope and images were captured with a NIKON microscopy camera coupled to the NIS software 4.45 (NIKON). Callose intensity quantification was performed according to Daudi et al. (2012). Callose intensity was calculated with ImageJ by counting the number of callose spots and assigning a value from 1 to 10 (10, saturated signal; 9, over 250 spots; 8, between 200 and 249 spots; 7, between 150 and 199 spots; 5, between 100 and 149 spots; 3, between 50 and 99 spots; 2, between 5 and 49 spots; 1, between 0 and 5 spots). Average callose measurements were based on at least 20 leaf pictures taken from 12 different seedlings. Trypan blue staining for cell death visualization was performed as previously described (Alcázar et al., 2009).

### Real-Time qPCR Expression Analyses

Total RNA isolated from 10-day-old seedlings was extracted using *TRIzol* reagent (Thermo Fisher). Two micrograms of RNA was treated with DNAse I (Invitrogen) and first-strand cDNA was synthesized using Superscript IV (Invitrogen) and oligo dT. Quantitative real-time PCR using SYBR Green I dye method was performed on Roche LightCycler 480 II detector system following the PCR conditions: 95°C for 2 min, 40 cycles (95°C for 15 s; 60°C for 30 s). qRT-PCR analyses were always performed on at least three biological replicates with three technical replicates each using *ACTIN2* (*At3g18780*) as the internal control gene. Relative expression was calculated by 2^–ΔΔCt^ method (Livak and Schmittgen, 2001). Primer sequences used for expression analyses are shown in Supplementary Table 1.

### *Pseudomonas syringae* pv. *tomato* DC3000 and *hrcC* Inoculation Assays

*Pseudomonas syringae* pv. *tomato* DC3000 (*Pst* DC3000) and *P. syringae* pv. *tomato* DC3000 *hrcC* (*hrcC*) bacteria were streaked on solid NYGA medium (5 g/L bacto peptone, 3 g/L yeast extract, and 20 mL/L glycerol, with 15 g/L agar for solid medium) containing 25 μg/ml rifampicin. Single colonies were transferred to liquid NYGA supplemented with 25 μg/ml rifampicin and grown overnight at 28°C. Bacterial suspensions were washed two times with water and suspended on 10 mM MgCl_2_ to OD_600_ = 0.2. Silwet L-77 was added to a final concentration of 0.04% (v/v) before spray inoculation of 3-week-old *Arabidopsis* plants. Leaves were harvested after 3 h and 72 h of pathogen inoculation for the determination of bacterial growth as described in Alcázar et al. (2010). At least three biological replicates were determined for each time point of analysis.

## Results

### Polyamine Levels in Response to *Pst* DC3000 and *Pst* DC3000 *hrcC* Bacteria

**FIGURE 1:**
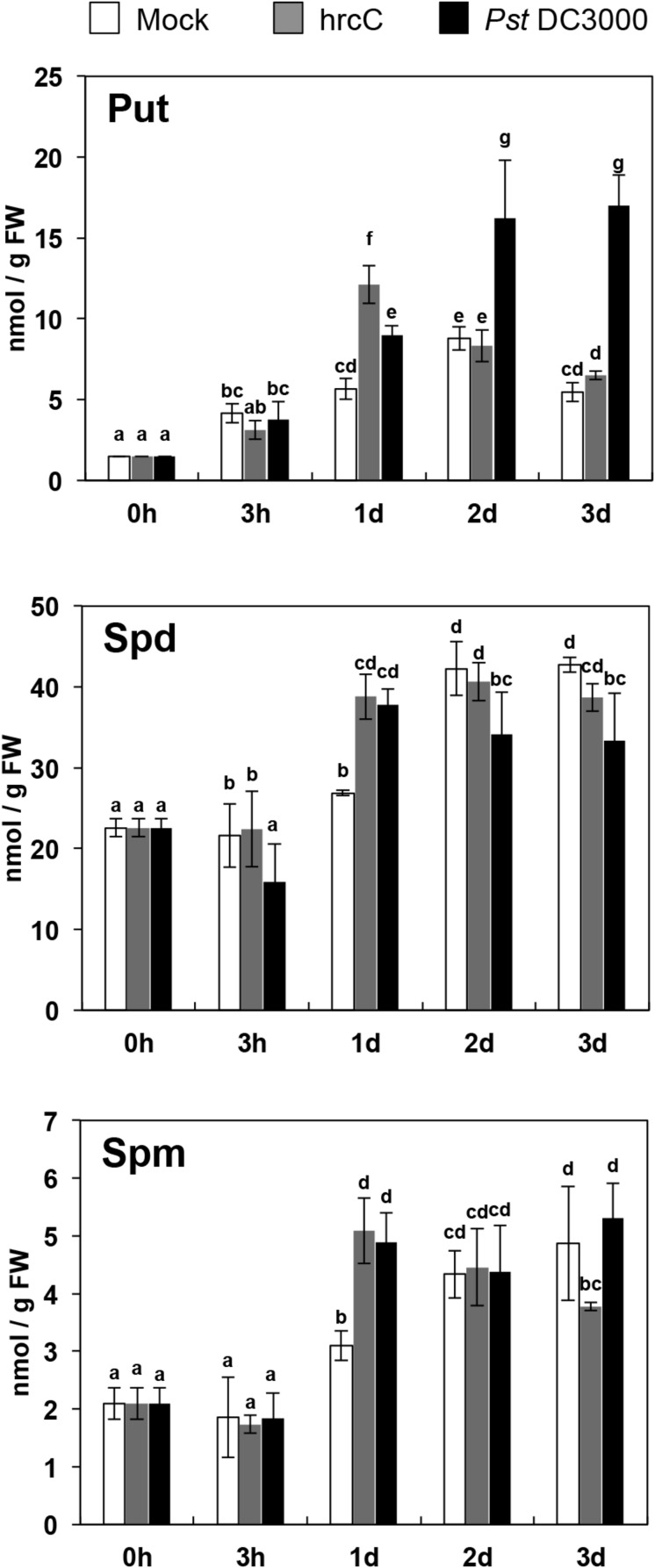
Polyamine levels in response to *Pst* DC3000 and *hrcC* inoculation. Levels of free putrescine (Put), spermidine (Spd), and spermine (Spm) in 3-week old *Arabidopsis* wild-type (Col-0) plants after 0 h to 3 days of spray inoculation with *Pseudomonas syringae* pv. *tomato* DC3000 (*Pst* DC3000), *Pst* DC3000 *hrcC* mutant (*hrcC*), or mock. Values are the mean of three biological replicates ± SD (standard deviation). Letters indicate values that are significantly different according to Student–Newman–Keuls test at *P* value <0.05.

The *P. syringae* pv. *tomato* DC3000 *hrcC* mutant (*hrcC*) is defective in the TTSS and mainly induces a PAMP-triggered response by failing to secrete defense-suppressing type III effectors into the plant cell (Yuan and He, 1996). In order to analyze the involvement of polyamines during PTI, we inoculated *Arabidopsis* wild type (Col-0) with *hrcC* and monitored the levels of free Put, Spd, and Spm for 3 days (Figure 1). One-day post-inoculation, the levels of Put, Spd, and Spm were 2.1-, 1.4-, and 1.7-fold higher in plants inoculated with *hrcC* than in mock inoculated plants (Figure 1). These results indicated that polyamines, and particularly Put, accumulated transiently in response to non-pathogenic *hrcC* bacteria, thus suggesting the participation of polyamines in the metabolic reprogramming induced during PTI.

In order to determine whether type III effector proteins suppress the changes in polyamine levels observed after *hrcC* inoculation, we determined Put, Spd, and Spm contents in plants inoculated with *P. syringae* pv. *tomato* DC3000 (*Pst* DC3000), which carries a functional TTSS (Figure 1). Compared to mocks, the Put levels increased up to 1.6- and 2.7-fold 1 and 2 days after inoculation with *Pst* DC3000, respectively. Spd and Spm levels also increased up to 1.4- and 1.6-fold 1 day post-inoculation. These results indicated that type III effectors delivered by *Pst* DC3000 do not suppress increases in polyamine levels triggered by *hrcC*. Rather, Put accumulation was higher in the strain provided with a functional TTSS.

### Determination of Apoplastic Polyamines

Some polyamines have been reported to accumulate in the apoplast of *Arabidopsis*, tobacco, tomato, and rice during defense (Yoda et al., 2009; Vilas et al., 2018). Under basal conditions (0 h), the levels of free polyamines in the apoplastic enriched fractions were undetectable. However, apoplastic Put and Spd contents remarkably increased after 24 h of *Pst* DC3000 and *hrcC* inoculation. The levels of Put remained high in *Pst* DC3000 but not in *hrcC* inoculated plants. Apoplastic Spm was not detectable in any treatment (Supplementary Figure S1). We concluded that Put and Spd accumulate in the apoplast in response to *Pst* DC3000 and *hrcC* inoculation. These data suggested that polyamines could trigger some defense response from the cell surface against bacterial infection.

### Polyamine Levels in Response to flg22

**FIGURE 2:**
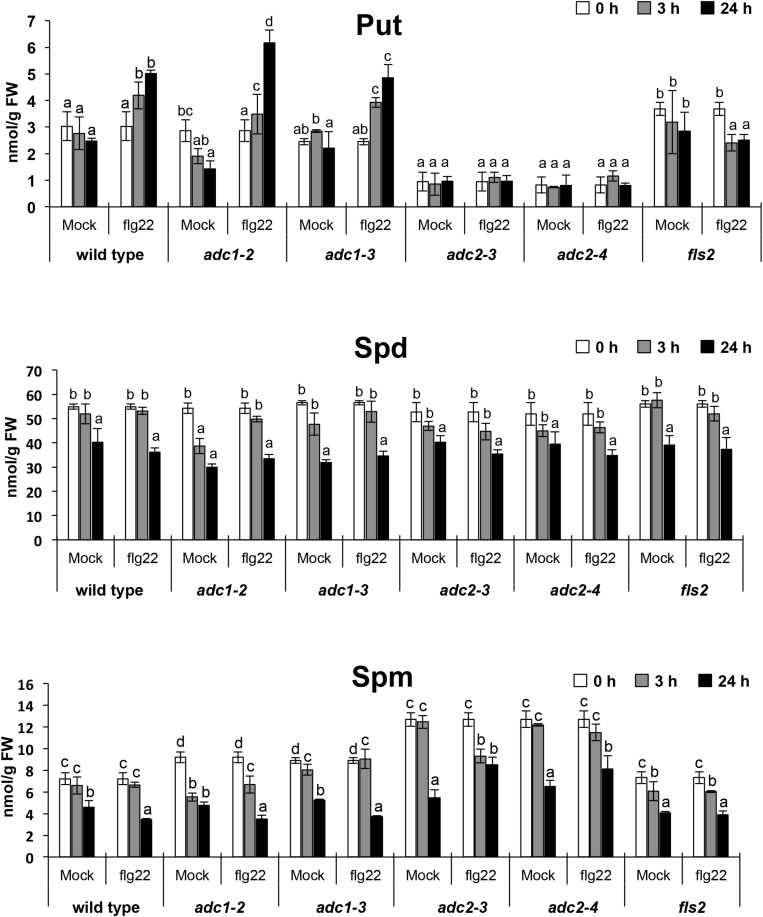
Polyamine levels in response to flg22 treatment. Levels of free putrescine (Put), spermidine (Spd), and spermine (Spm) in 10-day-old wild-type, *adc1-2*, *adc1-3*, *adc2-3*, *adc2-4* (Cuevas et al., 2008), and *fls2* (Zipfel et al., 2004) seedlings treated with 1 μM flg22. Seedlings were grown *in vitro* on a nylon mesh in ½ Murashige and Skoog media and transferred to the same media supplemented with 1 μM flg22 or mock for 24 h. Samples were harvested after 0, 3, and 24 h of treatment for polyamine analyses. Results are mean of three biological replicates ± SD (standard deviation). Letters indicate values that are significantly different according to Student–Newman–Keuls test at *P* value <0.05.

To further investigate the involvement of polyamines during PTI, we analyzed polyamine levels in response to the PAMP flg22. Free Put, Spd, and Spm levels were determined in wild type and *fls2* seedlings treated with 1 μM flg22 or mock (Figure 2). In the wild type, Put accumulated up to twofold in response to 1 μM flg22 treatment after 24 h. This increase was not evidenced in the *fls2* mutant (Figure 2), which indicated that Put accumulation triggered by flg22 was due to *FLS2*-dependent activation of PTI. The levels of Spd and Spm in seedlings treated with 1 μM flg22 did not exhibit significant changes compared with the mock control (Figure 2). Therefore, flg22 did not favor the synthesis or accumulation of Spd and Spm. However, increases in these polyamines were detected after 24 h of inoculation with *Pst DC3000* and *hrcC* bacteria (Figure 1). We suggest that other molecules produced by *P. syringae* (Xin and He, 2013) and perceived by the plant might trigger the synthesis of Spd and Spm in *Arabidopsis*.

### Involvement of *ADC* Isoforms in Put Biosynthesis Triggered by flg22

Arginine decarboxylase (ADC) catalyzes the conversion of arginine into agmatine, which is a limiting step in the biosynthesis of Put. In *Arabidopsis*, two *ADC* isoforms are found (*ADC1* and *ADC2*) that catalyze the same enzymatic reaction (Alcázar et al., 2006). To analyze the contribution of each isoform to Put synthesis in response to flg22, we treated *arginine decarboxylase 1 (adc1-2*, *adc1-3)* and *arginine decarboxylase 2* (*adc2-3*, *adc2-4)* loss-of-function mutants (Cuevas et al., 2008) with 1 μM flg22 and quantified the polyamine levels between 0 and 24 h (Figure 2). In *adc2-3* and *adc2-4*, the basal level of Put was much lower than in the wild type (Cuevas et al., 2008) and Put content did not increase in response to 1 μM flg22. Conversely, in *adc1-2* and *adc1-3*, Put content increased to a similar extent as the wild type in response to 1 μM flg22 (Figure 2). These results indicated that Put accumulation in response to flg22 is mainly contributed by ADC2 activity. Therefore, ADC1 and ADC2 forms do not act redundantly during PTI.

### Callose Deposition but Not Cell Death Is Induced by Put

**FIGURE 3:**
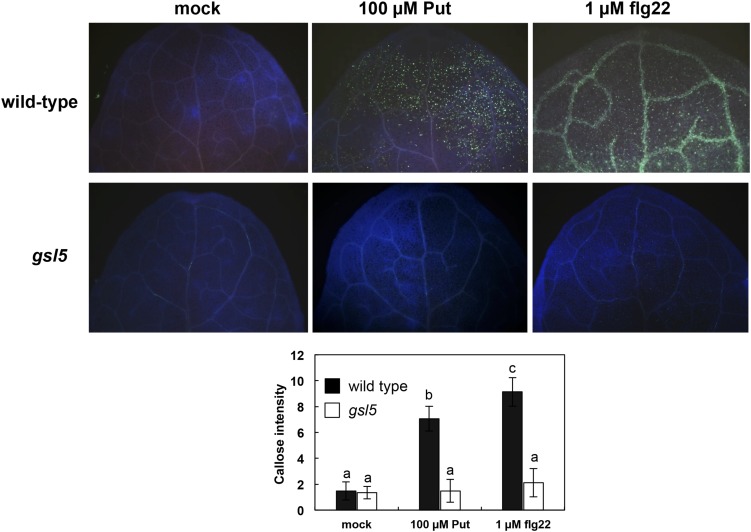
Callose deposition in response to Put treatment. Aniline blue staining of 10-day-old wild-type and *gsl5* loss-of-function mutants treated with Put or flg22 for 24 h. Leaves were drop inoculated with 5 μl of 100 μM Put or 1 μM flg22. Callose deposition was quantified based on image captions from at least 20 leaf pictures from 12 different seedlings per genotype and treatment. Letters indicate values that are significantly different according to Student–Newman–Keuls test at *P* value <0.05.

The increases in Put triggered by flg22 perception prompted us to investigate its potential role during PTI. Deposition of the (1,3)-β-glucan callose is induced in response to flg22, and it can be visualized by histochemical analysis based on aniline blue staining. We observed higher callose deposition in wild-type seedlings treated for 24 h with 100 μM Put or 1 μM flg22 than in seedlings treated with mock (Figure 3). Callose deposition induced by Put was compromised in the *glucan synthase like 5* (*gsl5*) mutant, which is defective in inducible callose accumulation upon wounding and biotic stress (Jacobs et al., 2003) (Figure 3). Conversely, callose deposition in response to flg22 was not obviously compromised in *adc1* or *adc2* mutants (Supplementary Figure S2). This indicated that flg22 responses are not impaired in *adc* mutants. To determine whether callose deposition triggered by Put was accompanied with cell death, we performed trypan blue staining in wild-type seedlings after 24 h of infiltration with 100 μM Put or mock (Supplementary Figure S3). Trypan blue staining did not reveal evident symptoms of cell death related with ETI in *Arabidopsis* leaves treated with 100 μM Put. These data indicated that Put infiltration does not induce HR in *Arabidopsis*.

### Expression of PTI Marker Genes in Response to Put

**FIGURE 4:**
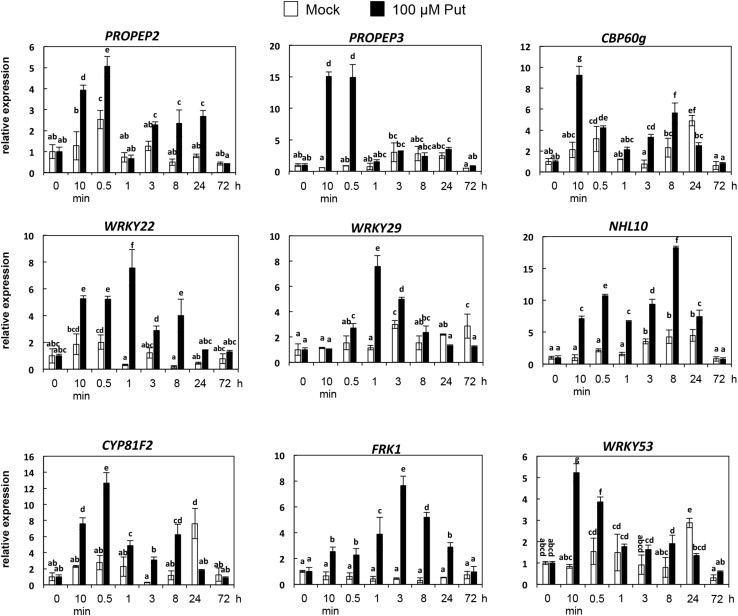
Expression analyses of PTI marker genes in response to 100 μM Put. Ten-day-old wild-type seedlings were treated with 100 μM Put or mock, and samples collected at 0 h, 10 min, 30 min, 1 h, 3 h, 8 h, 24 h, and 72 h post-treatment to analyze the expression of *PROPEP2*, *PROPEP3*, *CBP60g*, *WRKY22*, *WRKY29*, *NHL10*, *CYP81F2*, *FRK1*, and *WRKY53* as PTI marker genes by qRT-PCR. Seedlings were grown *in vitro* on a nylon mesh in ½ Murashige and Skoog media and transferred to the same media supplemented with the polyamine or mock up to 72 h. Expression values are relative to 0 h. Results are means of three biological replicates ± SD (standard deviation). Letters indicate values that are significantly different according to Student–Newman–Keuls test at *P* value <0.05.

**FIGURE 5:**
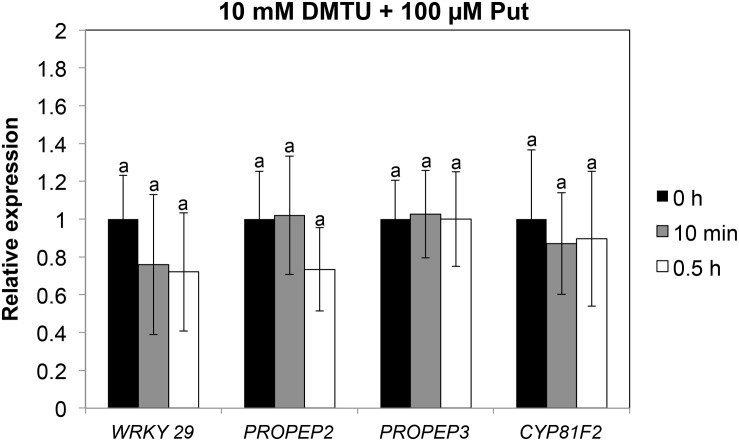
Expression analyses of PTI marker genes in response to 100 μM Put in the presence of 10 mM DMTU. Ten-day-old wild-type seedlings grown *in vitro* on a nylon mesh in ½ Murashige and Skoog media were transferred to the same media containing 10 mM DMTU for 3 h before treatment with 100 μM Put + 10 mM DMTU or 10 mM DMTU. Samples were collected after 0 h, 10 min, and 0.5 h of Put treatment for expression analyses of *WRKY29*, *PROPEP2*, *PROPEP3*, and *CYP81F2* by qRT-PCR. Expression values are relative to DMTU treatment at each time point of analysis. Results are the mean of three biological replicates ± SD (standard deviation). Letters indicate values that are significantly different according to Student–Newman–Keuls test at *P* value <0.05.

Accumulation of callose by Put suggested that PTI responses were activated by this polyamine. To further investigate this hypothesis, we analyzed the expression of several PTI marker genes (*PROPEP2*, *PROPEP3*, *CBP60g*, *WRKY22*, *WRKY29*, *WRKY53*, *CYP81F2*, *FRK1*, and *NHL10*) (Huffaker and Ryan, 2007; Xiao et al., 2007; Denoux et al., 2008; Wang et al., 2009; Boudsocq et al., 2010; Cheng et al., 2013; Po-Wen et al., 2013; Shi et al., 2015) in wild-type seedlings treated with 100 μM Put or mock between 0 and 72 h (Figure 4). For most of the genes analyzed, their transcripts increased rapidly in response to 100 μM Put, with the highest expression peaks observed upon 10 min to 1 h of treatment (Figure 4). These results indicated that Put induces transcriptional changes consistent with activation of PTI. Because Put can be oxidized by amine oxidases, we then studied whether transcriptional responses were compromised in the presence of the H_2_O_2_ scavenger dimethylthiourea (DMTU). For this, we determined the expression of *WRKY29*, *PROPEP2*, *PROPEP3*, and *CYP81F2* (Huffaker and Ryan, 2007; Denoux et al., 2008; Cheng et al., 2013) in wild-type seedlings treated or not with 100 μM Put in the presence of 10 mM DMTU (Figure 5). The increase in the transcript levels of these genes triggered by Put was compromised in the presence of DMTU (Figure 5). We concluded that H_2_O_2_ production is required for Put-triggered transcriptional up-regulation of PTI marker genes.

### Expression of PTI Marker Genes in Response to Put in *atrboh D*, *atrboh F*, and *atrboh D*/*F* Mutants

**FIGURE 6:**
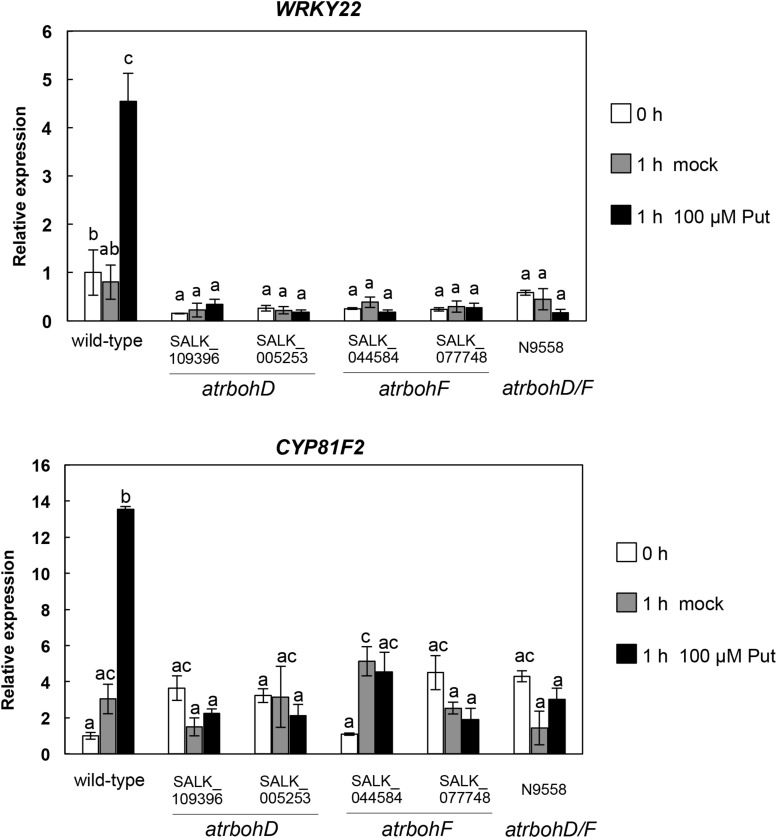
Expression analyses of PTI marker genes in response to 100 μM Put in *rbohD*, *rbohF*, and double *rbohD/F* loss-of-function mutants. Ten-day-old wild-type, *atrbohD* (SALK_109396 and SALK_005253), *atrbohF* (SALK_044584 and SALK_077748), and double *atrbohD/F* seedlings were treated with 100 μM Put or mock as described in Figure 4 and samples were collected at 0 and 1 h post-treatment to analyze the expression of *WRKY22 and CYP81F2* by qRT-PCR. Expression values are relative to the wild type at 0 h. Results are means of three biological replicates ± SD (standard deviation). Letters indicate values that are significantly different according to Student–Newman–Keuls test at *P* value <0.05.

Plasma membrane RBOHD and RBOHF are important sources of ROS during plant–pathogen interactions (Kadota et al., 2015). To determine the contribution of these NADPH oxidases to changes in the expression of PTI marker genes induced by Put, we analyzed the expression of *WRKY22* and *CYP81F2* in *atrbohD* (SALK_109396C and SALK_005253C), *atrbohF* (SALK_034674 and SALK_077748), and double *atrbohD/F* loss-of-function mutants (Torres et al., 2002) treated with 100 μM Put or mock (Figure 6). In contrast with the wild type, up-regulation of *WRKY22* and *CYP81F2* expression by Put treatment was strongly compromised in *atrbohD*, *atrbohF*, and double *atrbohD/F* mutants (Figure 6). These results indicated that Put requires functional RBOHD and RBOHF NADPH oxidases for signaling.

### Disease Resistance to *P. syringae* pv. *tomato* DC3000 and *hrcC* in Put Treated Plants

**FIGURE 7:**
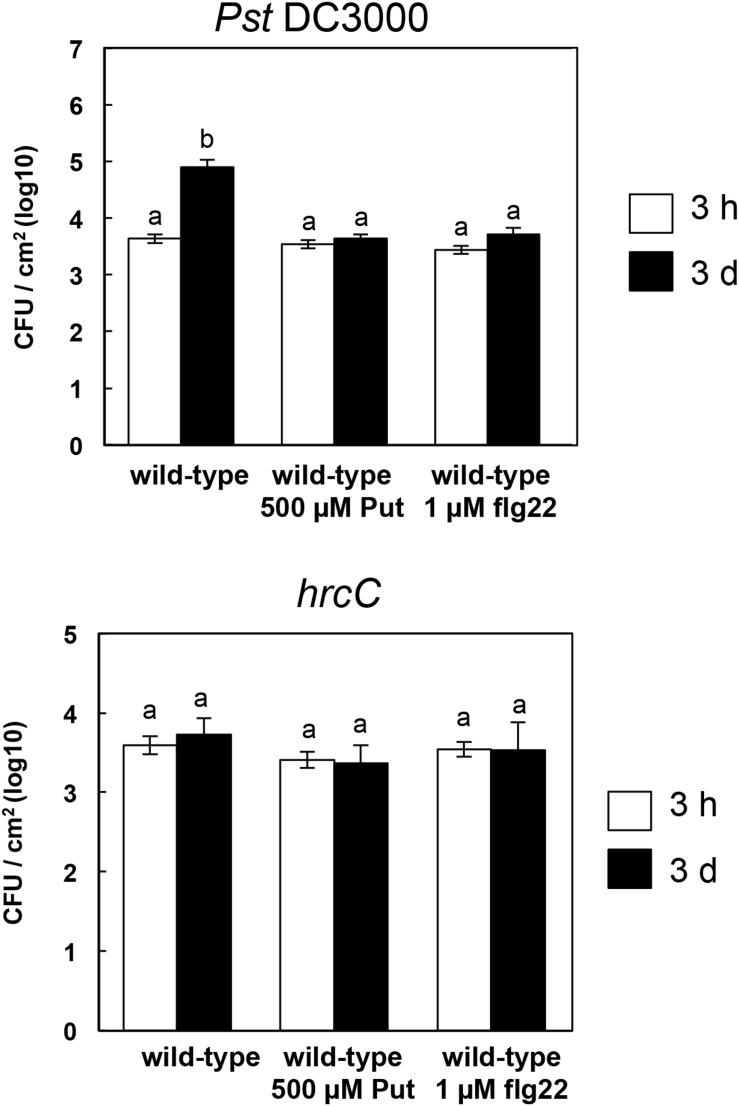
Growth of *Pseudomonas syringae* pv. *tomato* DC3000 and *hrcC* in wild-type pre-treated with Put or flg22. Three-week-old *Arabidopsis* plants were spray inoculated with 500 μM putrescine, 1 μM flg22, or mock 24 h before spray inoculation with *Pseudomonas syringae* pv. *tomato* DC3000 or *hrcC.* Bacterial counting was performed at 3 h and 3 days post-inoculation. Results are the mean of four replicates ± SD (standard deviation). Letters indicate values that are significantly different according to Student–Newman–Keuls test at *P* value <0.05.

So far, our data pointed to a role for Put contributing to amplify PTI responses. To analyze how this was translated into disease resistance, we performed pathoassays using *Pst DC3000* and *hrcC* bacteria in wild-type plants treated with 500 μM Put, 1 μM flg22 or mock. As shown in Figure 7, Put treatment limited the growth of *Pst* DC3000 to a similar extent as 1 μM flg22, whereas no differences were detected by inoculation with the non-pathogenic *hrcC* strain. We concluded that Put could be regarded as a priming agent contributing to basal defense responses against some pathogenic bacteria.

## Discussion

Plants are provided with an innate immune system that recognizes pathogens and activates defense responses. A first layer of the innate immunity involves the recognition of PAMPs, which are conserved signatures within a taxonomic group of pathogens. PAMPs include the flagellin peptide flg22, the elongation factor Tu (EF-Tu) peptides elf18 and elf26, lipopolysaccharides, fungal chitin, and peptidoglycan, among others (Boller and Felix, 2009). PAMPs induce the production of ROS, which participate in defense signaling and transcriptional reprogramming (Bigeard et al., 2015). During defense, ROS are predominantly generated by NADPH oxidases RBOHD and RBOHF (Torres et al., 2002; Kadota et al., 2015). However, other sources of apoplastic ROS include the activity of apoplastic peroxidases (Daudi et al., 2012) and amine oxidases (Cona et al., 2006). The different sources of ROS might be related to the necessity of specific ROS synthesis at different stages of the defense response (Cona et al., 2006). In *Arabidopsis*, the copper-containing amine oxidases (CuAO) *ATAO1/AtCuAOβ* (*At4g14940*) and *CuAO1/AtCuAOγ1* (*At1g62810*) have been localized in the apoplast (Moller and McPherson, 1998; Planas-Portell et al., 2013), whereas PAO enzymes have been found in the cytosol and peroxisomes (Tavladoraki et al., 2006; Kamada-Nobusada et al., 2008; Moschou et al., 2008; Takahashi et al., 2010; Ahou et al., 2014; Tiburcio et al., 2014). The apoplastic CuAOs preferentially catalyze the oxidation of Put (*ATAO1*) or Put and Spd (*CuAO1*) (Moller and McPherson, 1998; Planas-Portell et al., 2013), consistent with the occurrence of these polyamines in extracellular fluids (Yoda et al., 2009). Interestingly, *CuAO1* expression is induced by flg22 treatment (Planas-Portell et al., 2013), which suggests its participation in PAMP-triggered ROS signaling. The involvement of CuAO activities in the defense response of incompatible plant–pathogen interactions has previously been documented. In the incompatible interaction between barley and the powdery mildew fungus *B. graminis* f. sp. *hordei*, the levels of Put, Spd, as well as diamine oxidase and PAO activities were shown to increase and to contribute to defense through H_2_O_2_ production, leading to cell wall cross-linking of polysaccharides and proteins (Cowley and Walters, 2002). In chickpea, inhibition of CuAO activity was associated with decreased defense capacity against the necrotrophic fungus *Ascochyta rabiei* (Rea et al., 2002). The amount of free polyamines in the apoplast seems to be a limiting factor for CuAO activity (Rea, 2004). Indeed, it has been proposed that under stress conditions, polyamine excretion is activated in plant cells (Yoda et al., 2003). Consistent with this, the levels of apoplastic Put and Spd increase in response to avirulent *Pst* DC3000 *AvrRPM1* inoculation in *Arabidopsis* (Yoda et al., 2009).

Despite the growing body of evidence that shows the involvement of polyamines in defense, few studies have focused on the involvement of polyamines during PTI. In this work, we show that Put synthesis is stimulated by *Pst* DC3000 *hrcC* inoculation (Figure 1), a TTSS defective bacteria strain that mainly triggers a PTI response by failing to secrete effectors (Yuan and He, 1996). Consistent with this, Put level also increased by treatment with the purified PAMP flg22 (Figure 2). These data suggested that polyamines are part of the metabolic reprogramming response during PTI. Interestingly, inoculation with *Pst* DC3000, which carries a functional TTSS and can deploy effectors into the plant cell (Xin and He, 2013), did not suppress the increase in polyamine levels observed with *hrcC*. Rather, polyamine levels became higher (Figure 1). These results indicate that *Pst* DC3000 effectors are unlikely to suppress polyamine pathway activation. Rather, effectors might be promoting agents in polyamine biosynthesis. For example, the ADC1 isoform from *Capsicum annuum* is targeted by the *AvrBsT* effector from *Xanthomonas campestris* pv. *vesicatoria*. Their co-expression in *Nicotiana benthamiana* leaves promotes polyamine biosynthesis, thus leading to enhanced cell death and H_2_O_2_ production (Kim N.H. et al., 2013). However, it is not known whether Arabidopsis ADC isoforms might be targets of bacterial effectors. In Arabidopsis, the *ADC2* isoform is the major contributor to Put synthesis in response to flg22 (Figure 2). Consistent with this, Kim S.H. et al. (2013) showed that the *adc2* mutant (SALK_073977) in *Arabidopsis* compromises resistance to *Pst* DC3000, which can be rescued by infiltration with 2 μM Put.

The Put accumulation triggered by flg22 and *hrcC* prompted us to investigate the role of this polyamine during PTI. Interestingly, we found that exogenously supplied Put induces callose deposition in *Arabidopsis* seedlings (Figure 3). The formation of callose deposits is a typical physiological response of PTI. Callose is synthesized at the cell wall by callose synthases. The *Arabidopsis* genome contains 12 callose synthase (*CalS*) genes, also referred to as Glucan synthase-like (*GSL*) (Ellinger and Voigt, 2014). Among them, *GSL5* (*PATHOGEN MILDEW RESISTANCE 4*, *PMR4*) is required for wound and papillary callose deposition (Jacobs et al., 2003). We found that callose deposition induced by Put supply was compromised in the *gsl5* (*pmr4*) loss-of-function mutant (Jacobs et al., 2003) (Figure 3). To further investigate the involvement of Put during PTI, we selected a number of PTI marker genes based on previous reports (Huffaker and Ryan, 2007; Xiao et al., 2007; Wang et al., 2009; Boudsocq et al., 2010; Cheng et al., 2013; Po-Wen et al., 2013; Shi et al., 2015). Exogenously supplied Put rapidly led to the up-regulation of PTI marker genes tested (Figure 4). Interestingly, such responses were suppressed in the presence of the H_2_O_2_ scavenger, DMTU (Tate et al., 1982) (Figure 5). Hydrogen peroxide is likely derived from amine oxidase activity, thus pointing to an important role for polyamine oxidation during the transcriptional response triggered by Put. Interestingly, up-regulation of PTI marker genes was also compromised in *atrbohD*, *atrbohF*, and double *atrbohD/F* loss-of-function mutants (Figure 6). These data indicate that plasma membrane NADPH oxidases are required for at least some transcriptional responses induced by Put. In tobacco, the NADPH oxidases RBOHD/F have been suggested to act upstream of apoplastic PAO during salt stress, contributing to cell death (Gémes et al., 2016). Our data indicate that *Arabidopsis* RBOHD/F are downstream of Put or act in a concerted manner with apoplastic CuAOs during PTI. Collectively, we observed that PAMPs (flg22) induce Put biosynthesis and that Put triggers responses compatible with PTI activation, which are ROS and RBOHD/F dependent. Hence, a positive feedback loop is proposed in which Put amplifies PAMP-triggered signaling through ROS production, leading to enhanced basal disease resistance against bacterial pathogens (Figure 7). In this regard, apoplastic Put could act similarly to damage-associated molecular patterns (DAMPs) triggering a ROS-dependent defense response (Choi and Klessig, 2016; Versluys et al., 2017).

Collectively, our results gain insight into mechanistic processes by which polyamines contribute to disease resistance in plants. Such type of analyses should contribute to pave the road for the uses of polyamines as potential priming agents in agriculture.

## Data Availability

All datasets generated for this study are included in the manuscript and/or the Supplementary Files.

## Author Contributions

CL and KA performed the research. CL and RA planned the experiments. CL, KA, AT, and RA analyzed the data. RA wrote the manuscript.

## Conflict of Interest Statement

The authors declare that the research was conducted in the absence of any commercial or financial relationships that could be construed as a potential conflict of interest.

## Footnotes

1 www.arabidopsis.info

2 www.anaspec.com
