# Supplementary material for: The Polyamine Putrescine Contributes to H2O2 and RbohD/F-Dependent Positive Feedback Loop in Arabidopsis PAMP-Triggered Immunity
Source: Front Plant Sci. 2019 Jul 16;10:894. doi: 10.3389/fpls.2019.00894 (PMC6646693; doi:10.3389/fpls.2019.00894)
Supplement: Supplementary file 1 [file Data_Sheet_1.pdf]

SUPPLEMENTARY MATERIAL

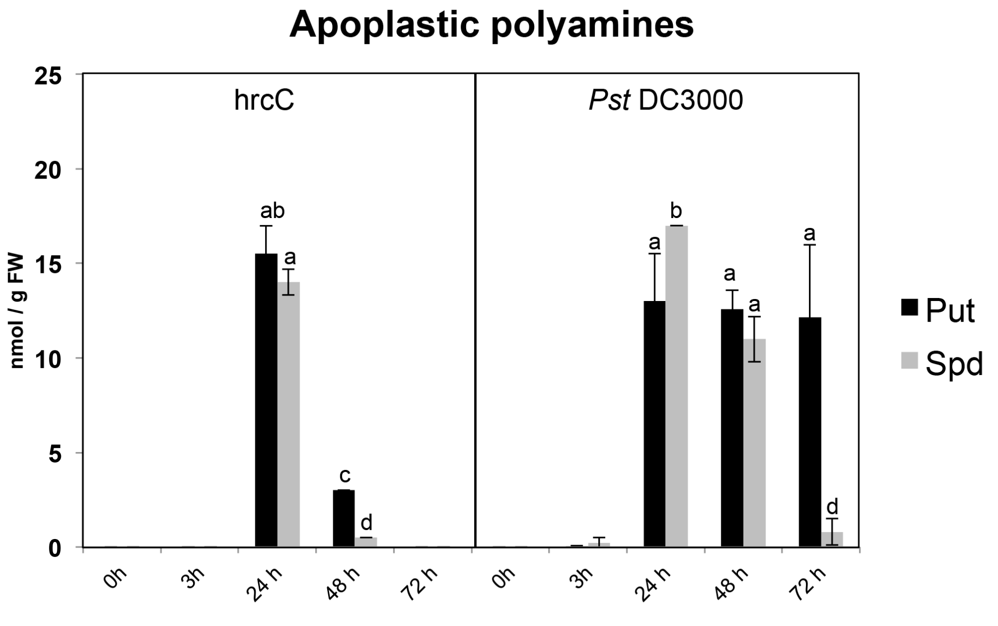

Figure S1. Levels of polyamines in apoplast enriched fluids of three-week old wild-type plants spray inoculated with *Pseudomonas syringae* pv. *tomato* DC3000 or *hrcC*. Samples for polyamine content determination were collected after 0 h, 3 h, 24 h, 48 h and 72 h of treatment. Values are the mean of three biological replicates ± SD (standard deviation). Letters indicate values that are significantly different according to Student–Newman–Keuls test at *P* value <0.05.FW, fresh weight.

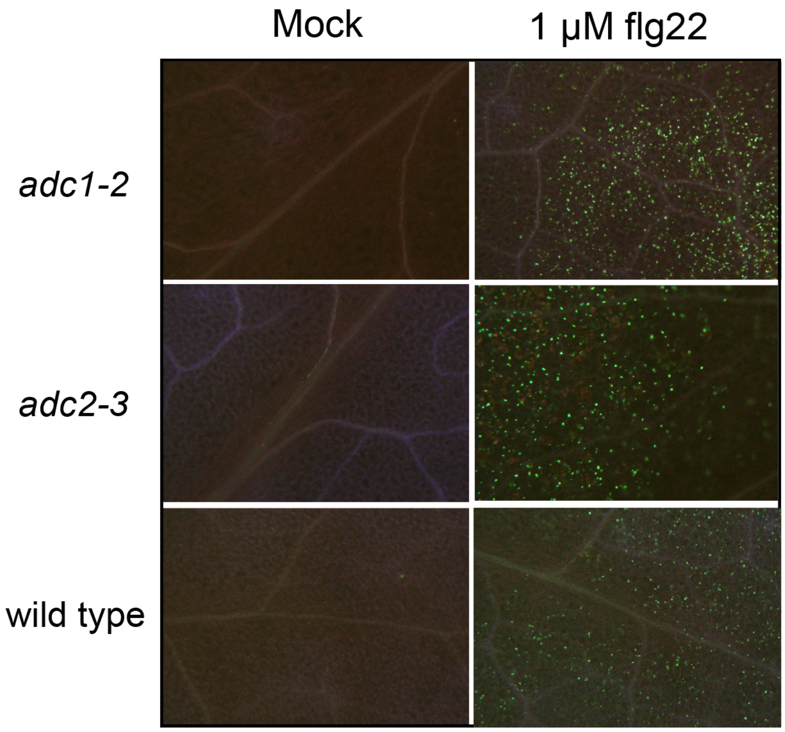

Figure S2. Aniline blue staining of callose deposits in *adc1-2*, *adc2-3* and wild-type seedlings treated with 1 µM flg22 or mock for 24 h. Treatments were performed as described in Figure 3.

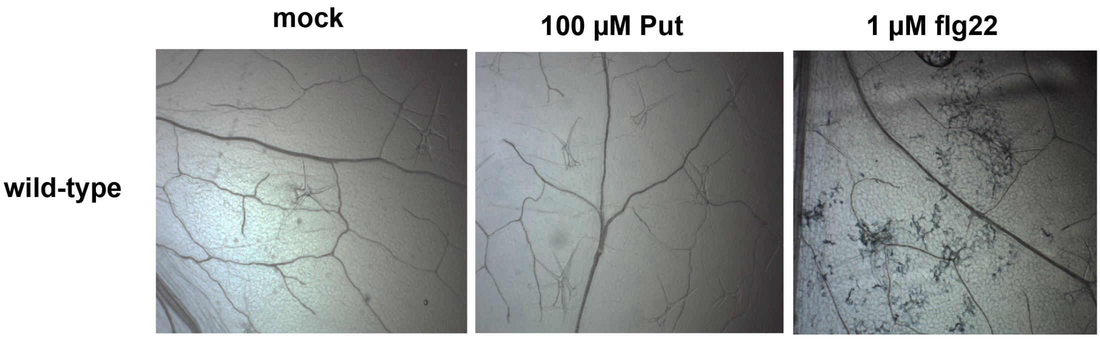

Figure S3. Trypan blue staining of 10-day old wild-type and *fls2* seedlings after 24 h of infiltration with 100 µM Put or 1 µM flg22. Treatments were performed in at least 12 different seedlings per genotype tested, showing similar results.

Supplementary Table 1. List of oligonucleotides and sequences used in this work.

Gene	Name	Forward (5' to 3')	Reverse (5' to 3')
At3g18780	Actin2	GATTCAGATGCCCAGAAGTCTTGT	TGGATTCCAGCAGCTTCC
AT5g64890	PROPEP2	AGAAAAGCCTAGTTCAGGTCGTC	CTCCTTATAAACTTGTATTGCCGC
At5g64905	PROPEP3	GTTCCGGTCTCGAAAGTTCATC	TCTTCCTCGCTGTGTGATGAC
At4g01250	WRKY22	CGTCCTCTTTCTCTCTCTGCTTCTTC	CCATGCCCAGACATCGGAGTTTA
AT4g23550	WRKY29	TTTCACCTTCGTTTTGCCTACC	CGAGCTCATCTAAGCCACTTGTC
AT4g23810	WRKY53	GGAGAAGCGACAAGACACCAGA	TATCCTTTGGCTTTTGGGTAATGG
AT2g19190	FRK1	GCCAACGGAGACATTAGAG	CCATAACGACCTGACTCATC
AT5g57220	CYP81F2	AAATGGAGAGAGCAACACAATG	ATCGCCCATTCCAATGTTAC
AT5g26920	CBP60g	AAGAAGAATTGTCCGAGAGGAG	GGCGAGTTTATGAAGCACAG
At2g35980	NHL10	TTCCTGTCCGTAACCCAAAC	CCCTCGTAGTAGGCATGAGC
